# Cultural attitudes and their impact on social exchanges, self-compassion, and mental health during pregnancy

**DOI:** 10.3389/fpsyg.2025.1668929

**Published:** 2025-11-27

**Authors:** Mona Al-Mutawtah, Mihela Erjavec, Hans-Peter Kubis

**Affiliations:** 1 School of Psychology and Sport Sciences, Bangor University, Bangor, United Kingdom; 2 Community Medicine-Clinical Psychology, Kuwait University, Kuwait City, Kuwait

**Keywords:** cultural attitudes, social exchanges, self-compassion, maternal mental health, pregnant women

## Abstract

**Introduction:**

Pregnancy is a crucial period for women’s physical and mental health, influenced by cultural attitudes and social relationships. This study explores how cultural orientations, social exchanges, and self-compassion interact to shape stress and depression during pregnancy, drawing on cultural psychology, social exchange theory, and stress-coping frameworks.

**Methods:**

A cross-sectional survey was conducted with 280 married Kuwaiti pregnant women recruited through convenience sampling. Data were analysed using structural equation modelling (SEM) to examine relationships among cultural attitudes (individualism and collectivism), social exchanges, self-compassion, perceived stress, and depression.

**Results:**

Individualism was positively associated with self-compassion, which in turn reduced stress and depression. Collectivism was linked to higher positive social exchanges, though these did not significantly buffer stress or depression. Negative social exchanges increased perceived stress, which mediated the relationship between negative social exchanges and depression. Planned pregnancy was associated with lower depression scores, while unplanned pregnancies correlated with higher negative social exchanges and greater stress. Higher energy levels significantly decreased depression.

**Discussion:**

Cultural attitudes play a dual role in maternal mental health. Individualism enhances self-compassion and reduces distress, while collectivism fosters positive support that does not necessarily alleviate stress or depression. These findings emphasise the importance of cultural tailored interventions that integrate self-compassion training with effective social support strategies to promote maternal well-being during pregnancy.

## Introduction

Pregnancy is a challenging life period, affecting physical and mental health in women, of whom 11% develop mental illness, predominantly depression, and 15% suffer from anxiety (Sharma and Sharma, 2022; Szemplińska et al., 2024). These challenges can affect the well-being of an individual depending on how they interact with their cultural and social environment, as these factors can either buffer or exacerbate psychological distress (Oyserman et al., 2002; Triandis, 1995). Social support has been identified as a key protective factor that can reduce pregnancy-related stress (Iranzad et al., 2014).

Beyond social support, broader cultural orientations such as individualism and collectivism may influence how women perceive and deal with the social challenges of pregnancy. Collectivism and individualism attitudes shape social behaviour, self-compassion, and mental health in unique ways (Donohue, 2021). Individualist cultures place a greater emphasis on personal autonomy, independence, and self-reliance (Stone-Romero and Stone, 2007). While this can foster resilience, it may also lead to smaller social networks, lower intentions to seek help from multiple sources, and higher levels of stress (Allik and Realo, 2004; Scott et al., 2004). This emphasis on self-reliance can be detrimental during pregnancy, a time when women often require increased support.

In contrast, collectivist cultures place a strong emphasis on social harmony, interdependence, and group identity (Gudykunst, 2003; Taylor et al., 2004; Power et al., 2010). While this can foster a sense of community and mutual support, it can also create pressure to conform to social norms and expectations, particularly regarding women’s roles during pregnancy and motherhood. For example, women may feel obligated to adhere to traditional gender roles, prioritise family needs over personal needs, and maintain social harmony, even if it comes at the expense of their own well-being. This pressure can be a significant source of stress (Donohue, 2021). Furthermore, collectivist societies may stigmatise mental health issues, making it difficult for pregnant women to seek support or openly discuss their struggles, leading to feelings of shame, guilt, and isolation. Traditional family roles or expectations can also contribute to stress, as pregnant women may feel obligated to fulfil these roles and expectations, such as intrusive questions (e.g., when will you get pregnant, annoyed comments about their body changes or pigmentations), unsolicited advice and outdated expectations from elderly relatives or gender preferences (Al-Mutawtah et al., 2023).

Cultural orientations can significantly influence a woman’s pregnancy experience, impacting how she perceives and responds to its inherent challenges. These orientations affect not only her general outlook but also her social interactions. Supportive social exchanges during this period can manifest themselves in various ways, such as providing emotional comfort and reassurance, relevant information and guidance, and practical assistance with daily tasks (Taylor et al., 2004). Moreover, empathy and non-judgmental acceptance further contribute to well-being (Ozbay et al., 2007). Conversely, negative social exchanges, such as critical remarks about physical changes or lifestyle choices, judgmental attitudes towards parenting decisions, and feelings of social isolation or exclusion (Al-Mutawtah et al., 2023), may amplify stress and detract from mental health (Lazarus and Folkman, 1984).

Adding to these factors, self-compassion, the act of treating oneself with kindness, understanding, and non-judgment during times of difficulty (Neff and Tóth-Király, 2022), is another critical factor influencing mental health. During the COVID-19 outbreak, self-compassion among pregnant women in Japan was shown to play a role in coping with pregnancy-related changes, with higher levels of self-compassion correlated with lower levels of difficult experiences, and buffering the negative impact of these experiences (Muramoto et al., 2022). A recent meta-analysis of 79 independent samples indicated that those collected from societies with higher levels of individualism were more likely to show a positive correlation between self-compassion and life satisfaction (Wang and Lou, 2022a; Wang and Lou, 2022b).

Few studies have explored the interrelationship between cultural orientation, social exchanges, and self-compassion in impacting stress and depression during pregnancy. For example, research has shown the importance of both social support and self-compassion during this period. A meta-analysis by Bedaso et al. (2021) found that low social support was significantly associated with an increased risk of depression and anxiety during pregnancy, highlighting the protective role of social connections. Furthermore, Abbaszadeh et al. (2023) demonstrated that self-compassion plays a mediating role between body image and the severity of anxiety and depression in pregnant women, suggesting that interventions aimed at increasing self-compassion could improve mental health outcomes. Therefore, self-compassion can be an important coping factor during pregnancy. Moreover, in our previous study, we investigated the perceptions of lifestyle changes and social support in pregnant women in Kuwaiti, considering cultural influences (Al-Mutawtah et al., 2023). The study utilised semi-structured interviews and reflexive thematic analysis. It explored pregnant Kuwaiti women’s perceptions of their female communities and found that while these communities offer support and connection, they also impose social pressure and expectations. Additionally, this research shed light on how traditional gender and family roles, as well as cultural norms, affect pregnant women’s experiences. Some women feel pressured to fulfil these traditional and cultural roles. For example, women blame themselves and feel guilty if they are not able to fulfil all their responsibilities towards the children or the household (Al-Mutawtah et al., 2023).

Building on this research and on the published literature, this study uniquely investigates the influence of cultural orientation on experience of negative and positive social exchange and their interrelationship with self-compassion for impacting stress and depression during pregnancy in light of the limited research on this topic in Kuwaiti context.

## Conceptual framework and hypotheses

### Conceptual framework

Cultural psychology can provide insight into how cultural values influence mental health outcomes (Markus and Kitayama, 1991). This inquiry is grounded in Hofstede’s (1980) and Triandis’ (1995) individualism–collectivism theory that argues that cultural orientation guides the fundamental structure of psychological priorities. Individualistic orientation emphasises the importance of autonomy and self-kindness, whereas collectivistic orientation emphasises relational harmony and communal support (Markus and Kitayama, 1991). Cross-cultural scholars propose that these cultural scripts shape coping strategies in different ways. For example, self-compassion may lead to better mental health outcomes in individuals who are more individualistic (Wang and Lou, 2022a; Wang and Lou, 2022b), while collectivism may lead to more prosocial interactions, which are known to ease relational stress (Kim et al., 2006).

In parallel, Lazarus and Folkman’s (1984) transactional model of stress highlights the importance of cognitive appraisal in shaping the impact of environmental challenges (e.g., an unplanned pregnancy) on mental health outcomes. This relationship is further nuanced by the stress-buffering hypothesis (Cohen and Wills, 1985). This hypothesis describes how social support can buffer or, conversely, how conflict can exacerbate stress reactivity, and recent meta-analyses support its conditional effectiveness across cultural and situational factors (Ozbay et al., 2007; Chu et al., 2010).

Connecting these perspectives together, Neff’s (2003) theory of self-compassion adds an important intrapersonal factor, theorising that self-kindness, mindfulness, and common humanity have the capacity to short-circuit maladaptive stress response systems, thereby providing a buffer against depression (Raes, 2011; Zessin et al., 2015). The effectiveness of those mechanisms, though, may depend on cultural context. Cross-cultural studies have shown, for example, that the benefits of self-compassion vary depending on the tendency toward individualism: independent self-enhancement is congruent with the Western notion of high relational worth (Kitayama et al., 2015), while relational coping may outweigh the intrapersonal focus that characterises collectivist contexts (Kuo, 2013).

At the heart of this framework is the conjecture that cultural orientations and social transactions are not only distal predictors of mental health but work through dynamic, stress-mediated pathways, conditioned by contextual vulnerabilities. Unplanned pregnancies, for example, can exacerbate stress through existential uncertainty, economic strain, or relational conflict (Foster et al., 2012; Abbasi et al., 2013), making a pregnant woman more vulnerable to depression. Planned pregnancies, in contrast, may elicit anticipatory social support and psychological preparedness which buffer stress appraisals (Biaggi et al., 2016).

By synthesising cultural, social, and stress coping theories, this study proposes an appropriate framework for how cultural, social, and self-compassion might be strategically mobilised to mitigate stress and depression in perinatal contexts. In consequence, the present study investigates a crucial omission in the literature by examining the interrelations between cultural attitudes, social exchanges, and self-compassion influencing mental health in pregnancy. In particular, it examines how individualism and collectivism, social support, and self-compassion interact to influence perceived stress and depression among pregnant women in Kuwait. The data were gathered via an online survey that included five questionnaires assessing cultural orientation, social interactions, self-compassion, stress, and depression. By linking these dimensions, this study aims to investigate how these variables interact with each other to influence maternal mental health during pregnancy.

### Hypotheses

*Hypothesis 1 (H1)*: Women with higher individualist attitude are more likely to have greater self-compassion, which leads to lower experience of perceived stress and depression.

*Hypothesis 2 (H2)*: Women with higher self-compassion report less perceived stress and depression levels.

*Hypothesis 3 (H3)*: Participants with higher collectivist attitude are more likely to have higher positive social support experiences, which lead to lower experience of perceived stress and depression.

*Hypothesis 4 (H4)*: Stress mediates the effect of negative social support experience on depression.

*Hypothesis 5 (H5)*: Pregnancy type contributes differentially to the experience of stress, with unplanned pregnancies being linked with a higher experience of negative social support and perceived stress, while planned pregnancies are linked with a lower level of perceived stress.

## Materials and methods

### Data collection and measures

The data collection took place June 19, 2024, to November 13, 2024. The study initially recruited 455 married Kuwaiti pregnant women. In Kuwaiti culture, all pregnant women are required to be married, as it is illegal to be pregnant outside of marriage. However, 175 participants were excluded from the study due to incomplete data (78 only completed the first scale; 69 stopped after the first two; and further 28 after the first three scales). The remaining 280 participants were included in the final sample. There were no significant differences across the scale scores between the participants who did not complete all of the measures and those who did {individualism [*F* (2,443) = 1.33. *p* = 0.267], collectivism [*F* (2,444) = 1.11, *p* = 0.331], negative social exchanges [*F* (1,366) = 0.70, *p* = 0.402], and positive social exchanges [*F* (1,371) = 0.17, *p* = 0.683]}. Chi-square tests show that there is no relationship between finishing the survey and factors like education, health status, or pregnancy characteristics. These checks confirm that excluding participants who did not complete all measures is unlikely to create bias in the results obtained from the sample of completers. The final sample size for the present study is in alignment with previous research conducted among pregnant women, which ranged from 111 to 375 (Fathnezhad-Kazemi et al., 2021; Kang and Yang, 2022; Raghuveer and Haleema, 2023; Rashan et al., 2021; Rastegari et al., 2023).

Table 1 shows the demographic characteristics of the participants. The final sample included 280 pregnant women (mean age = 31.53 years; *SD* = 4.98). Overall, 71.4% of participants had an undergraduate degree, and 51.8% were employed. Gestational age distribution was 24.6% in first trimester, 40.7% in the second and 34.6% in the third; and 39.3% of the pregnancies were unplanned. To maintain cultural consistency, the study was restricted to Kuwaiti citizens. There were no restrictions regarding age or pregnancy trimester. Data was collected between June and November 2024 via an online survey administered through the Qualtrics platform. Online data collection has several advantages; however, it is important to recognize its potential limitations, especially in terms of representation. For instance, individuals without internet access may not be included in the study, which may result in an underrepresentation of these groups. Future research could mitigate these limitations by using multiple recruitment methods, such as flyers or partnerships with community organizations, to reach a broader group of respondents. The survey, delivered in Arabic language, included six questionnaires and took approximately 30 min to complete. The decision to administer the survey in Arabic was based on the need to ensure clarity and comprehension, as participants were not expected to be familiar with English terminology. The participants were provided with a written informed consent form at the beginning of the survey, which outlined the study’s purpose, confidentiality assurances, and their right to withdraw at any time. Only those who provided consent were permitted to participate in the survey.

**Table 1:** The demographic characteristics of participants.

Characteristics	*N* (%)
Education level	
Postgraduate	35 (12.5)
Undergraduate	200 (71.4)
Diploma	38 (13.6)
High school	7 (2.5)
Region	
Al-Ahmadi	22 (7.9)
Mubarak Al-Kabeer	44 (15.7)
Al-Jahra	46 (16.4)
Al-Asimah	80 (28.6)
Al-Farwaniya	30 (10.7)
Hawalli	58 (20.7)
Current employment	
Employed	145 (51.8)
Unemployed	32 (11.4)
Medical leave	103 (36.8)
Gestational age	
First trimester	69 (24.6)
Second trimester	114 (40.7)
Third trimester	97 (34.6)
Pregnancy type	
Planned	170 (60.7)
Unplanned	110 (39.3)

*N*, number of participants; %, percentage of total sample.

### Ethics

The study protocol was approved by the Bangor University Ethics and Governance Committee (number 2024-0285-4) and the Kuwait Health Authorities (number 2024-2595). The participants were provided with a written informed consent form that explained the study’s objectives, ensured confidentiality, and their right to withdraw at any time without giving any explanation. On completion, the participants were provided with links to support helplines in case of distress.

### The measures

1. Auckland Individualism and Collectivism Scale (AICS)-26 items: AICS is a reliable and valid tool for measuring collectivism and individualism across diverse populations and languages, making it a trustworthy tool for measuring these important cultural characteristics (Shulruf, 2023). The first author translated AICS into Arabic language and had it back translated into English by a professional translator. A back translation is intended to verify the accuracy and fidelity of the forward translation by ensuring that the original meaning and nuances are accurately preserved in the back translation (Alabdulaziz et al., 2020). The research team has reviewed the back-translated English version against the original English version; discrepancies were discussed with the professional translator, and improvements were then added to the forward-translated text. For item responses, a 6-point scale is used ranging from 1 (never) to 6 (always). Cronbach’s Alpha was computed for each construct to evaluate the internal consistency of the scales used in the study. The two subscales measuring cultural attitudes had high internal consistency, with the collectivism subscale containing 11 items (Cronbach’s α = 0.816) and the individualism subscale 15 items (α = 0.814).
2. Positive and Negative Social Exchange Experiences (PANSE): It is a tool consisting of two subscales (12 items on each subscale). The Positive Social Exchange scale measures different types of social support (information, emotional, practical and companionship) experienced over the last month. The Negative Social Exchange scale measures the frequency of experiencing unwelcome advice, unhelpful behaviour, and rejection from social relationships (Jiang et al., 2023). A 5-point scale is used to assess item responses, ranging from 0 (never) to 4 (very often). The first author translated this scale to Arabic and applied the same forward-backwards translation process described previously. Reliability of the social exchange measures was satisfactory, with positive social exchanges (α =  0.767, 12 items) and negative social exchanges (α = 0.772, 12 items).
3. The Arabic version of the Self-compassion Scale SCS-SF short form: It is a 12-item version of the original 26-item Self-Compassion Scale. The SCS-SF measures three main aspects of self-compassion: self-kindness versus self-judgment; common humanity versus isolation; and mindfulness versus over-identification. It uses a 5-point scale ranging from 1 (never) to 5 (almost always) for item responses (Hayes et al., 2016). The Cronbach’s Alpha value for the Arabic version is 0.86, which is considered very good (Alabdulaziz et al., 2020). In our study, self-compassion (α = 0.780, 12 items) had an acceptable reliability.
4. The Arabic version of the Perceived Stress Scale-10 item: It is one of the most widely used psychological instruments for measuring stress perception. Items were designed to capture respondents’ feelings of unpredictability, uncontrollability, and overburden (Lee, 2012). The item responses are rated on a 5-point scale ranging from 0 (never) to 4 (very often). According to Roof et al. (2019), the internal consistency of the Arabic version of PSS was good (α = 0.776, 10 items). In our study, the internal consistency was high (10 items, α = 0.868).
5. The Arabic version of 36-Item Short Form Survey Instrument (SF-36): This is widely validated and widely used to assess subjective quality of life (QOL) (Zhang et al., 2012). It contains 36 questions about quality 8 component scores, physical functioning, role limitations-physical, role limitations-emotional, energy/fatigue, emotional well-being, social function, bodily pain and general health. In our study, we were interested in energy /fatigue levels, so we just used this subscale. This subscale includes 4 items assessing feelings of energy, low mood, weariness and tiredness. The score ranged from 0 to 100, higher score reflects higher level of energy and lower level of fatigue. According to Coons et al. (1998), the Cronbach Alpha value of the fatigue/energy score in the Arabic version was 0.78. The internal consistency of fatigue/energy levels in our study was acceptable (4 items, α = 0.76).
6. The Arabic version of Patient Health Questionnaire-9 (PHQ-9): PHQ-9 is a nine-item self-assessment tool used to screen for depression. It addresses nine of the symptoms of major depressive disorder as defined by the DSM-IV. The respondents rate the frequency with which they have experienced these symptoms in the past 2 weeks on a scale from 0 (not at all/never) to 3 (nearly every day). The Cronbach Alpha of the Arabic version was 0.85, reflecting good internal consistency (Maroufizadeh et al., 2019). In the present study, PHQ (α = 0.847) also showed good internal consistency.

### Methods

#### Statistics

In the reporting of the data, the means and standard deviations were used to summarise continuous variables. All parameters were evaluated for normality and distribution using Shapiro–wilk test. As well as by examining skewness and kurtosis values. As general guideline, skewness and kurtosis values between −1.0 and +1.0 are considered acceptable (Mallery and George, 2000). The result in Table 2 indicated that individualism, self-compassion, and perceived stress met the assumption of normality according to the Shapiro–Wilk test (*p* > 0.05). However, collectivism, positive and negative social exchanges, energy/fatigue and depression significantly deviated from normality (*p* < 0.05). Cronbach’s Alpha was used to test the internal consistency of the measurement instruments (see Table 2).

**Table 2:** Summary of normality statistics and reliability.

Variable	Shapiro–wilk statistic (*p*-value)	Skewness	Kurtosis	Cronbach’s alpha
Individualism	0.994 (*p* = 0.109)	−0.039	−0.343	0.814
Collectivism	0.990 (*p* = 0.005)	0.156	−0.452	0.816
Self-compassion	0.992 (*p* = 0.105)	−0.087	0.254	0.780
Positive social exchanges	0.984 (*p* < 0.001)	−0.314	−0.269	0.767
Negative social exchanges	0.968 (*p* < 0.001)	0.507	−0.356	0.772
Perceived stress	0.992 (*p* = 0.077)	−0.120	0.145	0.868
Energy/fatigue	0.979 (*p* = 0.001)	0.307	−0.285	0.760
Depression	0.980 (*p* < 0.001)	0.384	−0.417	0.847

After conducting these preliminary analyses, structured equation modelling (SEM) with Maximum Likelihood (ML) estimation was conducted using AMOS (version 27) as a method for evaluating the conceptual framework developed in this study because it allows for the simultaneous testing of multiple relationships between the study variables, while correcting for measurement errors (Cao, 2023; Kline, 2016). SEM is particularly well-suited to mediation analyses, making it ideal for examining the indirect effects of self-compassion on mental health outcomes (MacKinnon, 2008). Moreover, this method can evaluate model fit and refine hypothesised relationships to provide the results that are both statistically and theoretically valid. The SEM analysis highlights protective factors and risk pathways that influence maternal mental health by incorporating direct and indirect pathways. The model had 15 variables—9 observed variables and 6 unobserved variables (error terms e1, e2, e3, e4, e5, and e6)—and was tested on 280 participants. To ensure adequate statistical power, we performed a post-hoc power analysis with the Satorra-Sarstedt method. Kumar et al. (2023) found that there was an average effect size of r = 0.32 for the relationship between self-compassion and depression in pregnant women. According to these findings, we used an expected effect size of 0.3, which resulted in an estimated power of 0.82.

## Results

### Descriptive analysis

An overview of the key study variables is presented in Table 3. These variables include individualism and collectivism attitudes, social exchanges, self-compassion, perceived stress, energy/fatigue, and depression scores.

**Table 3:** The variables (parameters) used in the present study.

Parameter	*N*	Minimum	Maximum	Mean	SD
Individualism attitude	280	33	83	61.8	9.9
Collectivism attitude	280	23	62	42.1	8.4
Positive social exchanges	280	8	48	32	8.5
Negative social exchanges	280	0	48	17.1	10.8
Self-compassion	280	18	60	39.5	6.6
Perceived stress	280	0	40	21.1	6.6
Energy/fatigue	280	0	100	35.6	20.1
Depression score	280	0	27	11.5	5.7

N, number of participants; SD, standard deviation.

The scores of the individualism attitude range from 33 to 83, with a mean of 61.8 (*SD* = 9.9). The results indicate a moderate tendency toward individualism, but with significant variability. The sample shows a moderate collectivistic attitude with scores ranging from 23 to 62, with a mean of 42.1 (*SD* = 8.4).

In terms of positive social exchanges, the range of this measure is 8 to 48, and the mean is 32 (*SD* = 8.5). This finding indicates that participants experience positive exchanges, although there is some variation. Regarding negative social exchanges, the score ranges from 0 to 48, with a mean of 17.1 (*SD* = 10.8), indicating that some participants have experienced negative social exchanges, while others report none.

Scores on self-compassion ranged from 18 to 60, with a mean of 39.5 (*SD* = 6.6), indicating moderate levels of self-compassion among participants. In terms of perceived stress, the data indicate that participants experience moderate stress ranging from 0 to 40, with a mean of 21.1 (*SD* = 6.6), though some report very low or very high levels of stress. Energy/fatigue scores ranged from 0 to 100, with a mean of 35.6 (*SD* = 20.1). Depression, the final parameter, ranges from 0 to 27, with a mean of 11.5 (*SD* = 5.7). This value indicates that, on average, participants experience mild to moderate depressive symptoms, with some reporting no symptoms and others experiencing severe symptoms.

### Structure equation modelling

**Figure 1:**
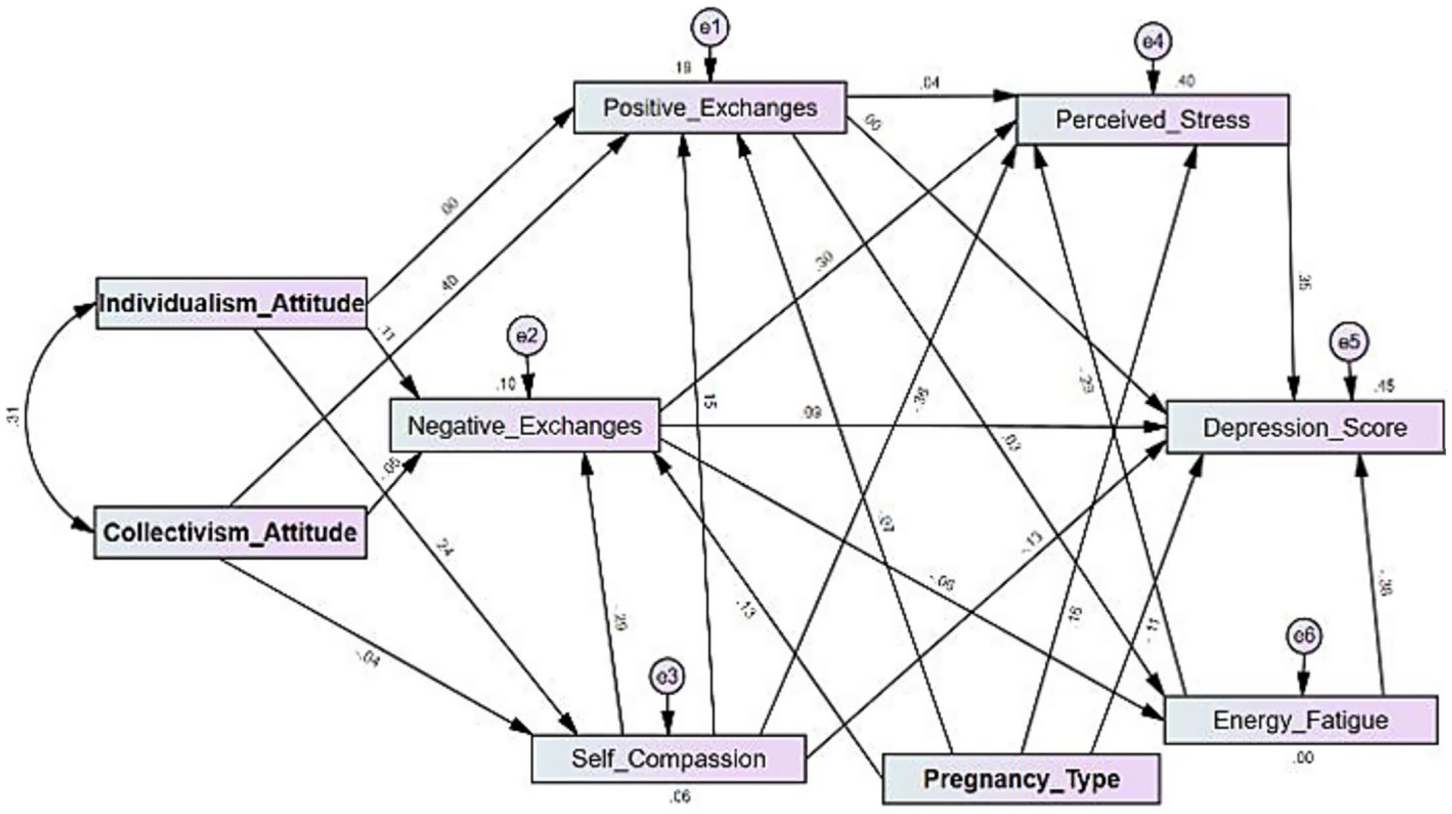
Path analysis.

Figure 1 shows the path analysis of the structural model. The Chi-square test (χ^2^ test): (12) = 101.384, *p* < 0.001. The model fit indices were as follows: CFI = 0.838 (acceptable fit ≥ 0.90, Hu and Bentler, 1999), TLI = 0.481 (acceptable fit ≥ 0.90, Hu and Bentler, 1999), RMSEA = 0.163 (90% CI: 0.135–0.193) (acceptable fit ≤ 0.80, Hu and Bentler, 1999), and GFI = 0.930 (acceptable fit ≥ 0.93, Cho et al., 2020). These indices suggest that the model fit is suboptimal, with the CFI, TLI and RMSEA failing to meet conventional cut-off criteria for good fit. Although the model fit was poor, we avoided post-hoc model modification since the primary goal was to test specific hypotheses about cultural attitudes and mental health during pregnancy, without improving the model’s fit. Further, there are some concerns with the ability to test the model using measures for the Kuwait population, as there may be validity and reliability issues with the measures. Due to this, we present the results of our a priori model, acknowledging its limitations and focusing on significant relationships observed, but with caution. It is important to mention that in this model, demographic and medical factors were not controlled, and these may have impacted results. SEM explained different extents of key psychological outcomes. The model explained 44.9% of the variance in depression scores (*R2* = 0.449), suggesting that nearly half the variation in depression levels can be attributed to the included predictors. Similarly, perceived stress showed significant variance explanation, with 40.5% (*R2* = 0.405) of its variability explained by the factors of the model. Positive social exchanges, on the other hand, were moderately predicted, explaining 19.4% of variance (*R2* = 0.194), while negative social exchanges explained only 9.9% (*R2* = 0.099). Finally, self-compassion showed the weakest prediction, accounting for only 5.5% (*R2* = 0.055) of its variance.

*H1*: Cultural attitudes, self-compassion, and mental health

Individualism attitude was significantly associated with increased self-compassion (β = 0.244, *p* < 0.001), while collectivism had no impact on self-compassion (β = −0.041, *p* = 0.501). In the model, self-compassion mediated the relationship between individualism and mental health outcomes; higher level of individualism was having a significant negative indirect effect on both perceived stress (β = −0.087, *p* < 0.001) and depression scores (β = −0.190, *p* < 0.001), via the influence on self-compassion. This fully supports Hypothesis 1, suggesting that individualism is positively associated with self-compassion, which lowers stress and depression through that pathway.

*H2*: Self-compassion, stress, and depression

Higher self-compassion directly reduced perceived stress (β = −0.357, *p* < 0.001) and depression score (β = −0.128, *p* = 0.012). Accordingly, this result support Hypothesis 2, suggesting that self-compassion serves as a protective factor against perceived stress and depression.

In terms of social exchanges, self-compassion was associated with increased positive (β = 0.15, *p* < 0.001) and reduced negative social exchanges (β = −0.472, *p* < 0.001).

*H3*: Collectivism, social exchanges, and mental health

Collectivism attitude was significantly associated with higher positive social exchanges (β = 0.401, *p* < 0.001), aligning with the first part of Hypothesis 3. However, positive social exchanges were not related to perceived stress (β = 0.037, *p* = 0.426) or depression (β = −0.004, *p* = 0.926). This suggests that collectivism attitude promotes positive social exchanges; yet these exchanges do not act as a buffer against perceived stress or depression in this sample.

*H4*: Stress as a mediator

Negative social exchanges were associated with increased perceived stress (β = 0.30, *p* < 0.001), and perceived stress, in turn, contributed to higher depression scores (β = 0.35, *p* < 0.001), supporting Hypothesis 4. Additionally, the indirect effects of negative social exchanges on both depression (β = 0.131, *p* < 0.001) and perceived stress (β = 0.016, *p* < 0.001) were significant. These indirect effects indicate that part of the effect of negative social exchanges on depression may work through heightened levels of perceived stress, which highlights stress as a key mediating pathway.

*H5*: Pregnancy type and mental health outcomes

Unplanned pregnancy was associated with increased negative social exchanges (β = 0.13, *p* = 0.024) and perceived stress (β = 0.16, *p* < 0.001); also, it indirectly increased depression (β = 0.084, *p* < 0.001) via perceived stress (β = 0.038, *p* < 0.001). By contrast, planned pregnancy was directly associated with reduced depression (β = −0.112, *p* = 0.015). These findings support Hypothesis 5.

### Energy/fatigue and mental health

Higher energy and lower fatigue levels had a significant negative direct (β = −0.381, *p* < 0.001) and indirect (β = −0.102, *p* < 0.001) effect on depression by lowering perceived stress, emphasizing its role in protecting against depressive symptoms.

## Discussion

The study examined the relationship between cultural orientations, social exchanges, self-compassion, and mental health outcomes among pregnant women in Kuwait. The women who took part were well-educated and representing varied gestational stages, with almost 40% of the pregnancies being unplanned. Cultural homogeneity was prioritised (only Kuwaiti nationals participated) to isolate the effects of societal norms, where familial and communal expectations have a significant impact on the experiences of women during pregnancy. The findings reveal distinct pathways through which cultural attitudes, pregnancy type, and self-compassion are associated with mental health outcomes among pregnant women in Kuwait. However, the results should be interpreted cautiously due to a poor fit of the model. Poor model fit can lead to biased parameter estimates (path coefficients) and underestimated standard errors. Consequently, there may be an increased risk of Type I errors (false positives) when interpreting the significance of individual pathways.

In our model, individualism had a significant effect on self-compassion but exerted no direct influence on positive social exchanges. This suggests the dual nature of individualism, fostering personal resilience and self-sufficiency without inherently prioritizing interpersonal relationships (Wang and Lou, 2022a). It is possible that individualism orientation fostered our participants’ self-compassion, in accordance with the theory proposed by Neff (2003), which emphasises self-kindness and emotional resilience. Further, self-compassion was found to be a protective factor directly impacting perceived stress and depression. Our findings that self-compassion protects against perceived stress and depression are supported by prior research. Kumar et al. (2023) reported that greater self-compassion during pregnancy was associated with a reduction in depression symptoms, mainly due to an increase in emotional resilience. Thus, self-compassion plays a fundamental role in enhancing emotional coping mechanisms, thereby buffering psychological distress—consistent with the protective effects we observed in our model.

In our sample, collectivism attitude was associated with positive social exchanges, which may reflect the importance of interdependence and mutual support in collectivist cultures (Vaidyanathan et al., 2013; Krys et al., 2022). This result agrees with Knyazev et al. (2017), who suggested that collectivist attitudes are associated with higher social cohesion and emotional support. However, these positive exchanges did not reduce stress, which goes against Cohen and Wills' (1985), stress-buffering hypothesis. It is possible that this paradox arises from the dual role of collectivist networks, in which they serve as both a source of support and obligations to conform to norms. Furthermore, Kuwaiti culture may involve strong expectations of deference to elders and adherence to traditional values. This can create stress for pregnant women even when receiving social support. According to Al-Mutawtah et al. (2025), Kuwaiti women reported experiencing pressure from their female community to conform to ideal expectations and adhere to traditional family and gender roles. For example, they may feel obligated to follow traditional (and sometimes harmful or unwanted) health advice from older relatives, even when they disagree with it or it conflicts with modern medical knowledge. This pressure to respect elders and conform to tradition, even when it is detrimental to their own well-being, may turn well-intentioned support into a source of stress. Additionally, there are some unique limitations to our analysis. We used a self-report measure of stress which is subject to various social desirability biases. Another possible explanation is that, despite the fact that the researchers followed the “forward-backward translation” process to ensure linguistic and conceptual accuracy, there may be cultural nuances in Kuwait that influence collective attitudes differently than in Western societies, including the role of extended families and religious values. This may be why, in our sample, on average, women endorsed collectivist values less than the individualist ones. On the other hand, the coexistence of individualistic and collectivist perspectives (covariance = 25.268, *p* < 0.001) suggests that these attitudes may mutually reinforce each other, promoting a balance between self-reliance and social interconnectedness in shaping behaviour. This may be in line with Kuwait’s cultural norms that reflect a hybrid approach which emphasises both personal independence and community obligations, influencing the way individuals interact with one another. However, the current study did not directly examine the impact of this synergy on mental health outcomes. Future research should explore whether this coexistence contributes to stress and depression. For example, the simultaneous endorsement of individualistic and collectivistic values may create conflicting expectations for women, leading to internal conflict and increased stress. In contrast, a balance between these values may be able to facilitate coping by allowing women to draw on both personal resilience and social support.

Consistent with Lazarus’s transactional model, stress served as a key mediator in our model, connecting negative social exchanges to depression. This finding is also consistent with Archuleta et al. (2023), who found that negative social exchanges significantly contribute to stress perceptions, explaining 35–43% of variance in stress measures in young and middle-aged adults. It is possible therefore that negative exchanges, such as conflict or criticism, heighten emotional distress particularly during pregnancy.

Regarding pregnancy type, unplanned pregnancies were significantly associated with elevated negative social exchanges and increased perceived stress, aligning with the transactional stress model (Lazarus and Folkman, 1984). These relationships were associated with depression, both directly and indirectly through stress. Our findings are consistent with previous research linking unplanned pregnancies with higher rates of depression, anxiety, and stress among pregnant women (Alsafar et al., 2022; Ermiati et al., 2022). In Saudi Arabia, for example, 35.3% of women with unplanned pregnancies experienced mild to severe depression, compared to 7.5% of those with planned pregnancies. Additionally, 46.6% of women with unplanned pregnancies reported mild to severe anxiety, as compared with 23.7% of women with planned pregnancies (Alsafar et al., 2022). Likewise, in other cultural contexts, young unmarried women with unplanned pregnancies reported high levels of anxiety and depression, with an adjusted odds ratio of 6.38 when compared to women with planned pregnancies (Fubam et al., 2019). The indirect effects of unplanned pregnancies on depression scores highlight its importance as a significance risk factor, necessitating targeted interventions for those women. Due to the model fit, the significant effects of pregnancy type on depression, anxiety, and stress should be viewed as provisional and not definitive. Conversely, in our sample, planned pregnancies were associated with reduced depression, possibly due to enhanced social support. This result is in accordance with a meta-analysis that determined that planned pregnancies significantly reduce the risk of antenatal depression, with an adjusted odds ratio of 0.45 (Putri et al., 2023).

In our sample, higher energy levels (lower fatigue) acted as protective buffers against depression, highlighting the importance of physical well-being in reducing the risks associated with mental health. Chou et al. (2008) have reported that fatigue, commonly caused by physical strain and hormonal changes during pregnancy, is associated with impaired emotional resilience and increased vulnerability to psychological distress. Based on these findings, interventions can be utilised to maintain or enhance levels of energy in pregnant women. Chan et al. (2019) conducted a systematic review of 29 articles and concluded that physical activity interventions can alleviate pregnancy-related pain and psychological symptoms, such as depression. Additionally, these interventions can improve the quality of life of pregnant women, underscoring the comprehensive advantages of sustaining an active lifestyle during pregnancy. According to a recent meta-analysis of seven studies by Zhang et al. (2024), exercise interventions have a significant impact on antenatal depression symptoms in pregnant women, with an effect size of −0.41. In particular, static exercise was more effective than dynamic exercise, and interventions before 20 weeks’ gestation yielded better results. Additionally, longer interventions, which exceeded the duration of a trimester, were found to have a more positive effect. It is, however, important to note that Kuwaiti cultural norms often discourage women from exercising during pregnancy, suggesting the need for culturally adapted interventions that promote safe movement without contravening local customs (Al-Mutawtah et al., 2023). For example, a culturally adapted form of cognitive behavioural therapy (CBT) incorporating Islamic principles, such as patience, trusting God, remembrance, and self-accountability, might enhance cultural fit and therapeutic alliance. In pregnant women, research on the effectiveness of these adaptations is limited at present; however, they have shown promise in other populations (e.g., Sabki et al., 2019; Subhas et al., 2021; Ariff and Syed, 2025).

## Limitation

This quantitative study employed a cross-sectional design, collecting data at a single point in time, which prevents the establishment of causal relationships between variables (Shadish et al., 2002). For example, while negative social exchanges were associated with higher depression, causality cannot be inferred; the relationship could be bidirectional or influenced by unmeasured confounding variables (Maxwell and Cole, 2007). Future longitudinal studies are needed to examine how social support dynamics, cultural orientations, self-compassion, and mental health evolve throughout pregnancy and into the postpartum period. Tracking these variables over time would provide a more robust understanding of their interplay and identify critical periods for intervention. Furthermore, while we collected data regarding demographic and medical factors, such as medical or psychological illnesses, these variables were not included in our primary SEM analysis. Due to this, we cannot exclude the possibility that these factors have influenced the observed relationship. Future research needs to investigate whether these factors play a role in moderating the relationship between cultural attitudes, self-compassion and mental health during pregnancy. Future research could also explore using more robust estimation methods to violations of normality for confirming our findings, such as robust maximum likelihood, to confirm the robustness of our results. Additionally, the convenience sampling through online distribution limits the representativeness of the sample and the generalisation of the statistical findings to the broader population of pregnant women in Kuwait (Etikan et al., 2016). Moreover, the reliance on self-report measures may have introduced biases such as social desirability or recall bias, which could influence the accuracy of the reported relationships; future research could consider including objective measures or data from multiple sources to reduce these biases. Finally, it is essential to acknowledge that the model fit was suboptimal, as the CFI and TLI failed to meet the conventional cut-off criteria for a good fit; therefore, the results should be interpreted with caution. It is possible that future research can refine the model by exploring additional relevant variables, non-linear relationships, or alternative modelling techniques, leading to a better-fitting and more thorough understanding of these complex relationships. However, we did not engage in post hoc model modification based on standardised residuals or modification indices, as such modifications can capitalise on chance and lead to overfitting of the data (Tomarken and Waller, 2003; Zheng and Bentler, 2023). By presenting our results with this limitation, the observed relationships provide valuable insights.

## Conclusion

Overall, this study illustrates the dual nature of Kuwaiti cultural values: individualism fosters self-compassion without eroding social cohesion, whereas collectivism strengthens or increased positive social support, but introduces obligations that may counterbalance the positive effects of this support. Stress plays a mediating role and emphasises the importance of social interactions to mental health outcomes. According to these findings, interventions that harmonise self-compassion practices with communal support systems, such as the incorporation of mindfulness techniques into prenatal care, may be helpful. A culturally adapted approach, addressing both the protective and oppressive aspects of collectivism, may enhance resilience while preserving social norms.

## Data Availability

The raw data supporting the conclusions of this article will be made available by the authors, without undue reservation.
